# Ultrastructural and biochemical classification of pathogenic tau, α-synuclein and TDP-43

**DOI:** 10.1007/s00401-022-02426-3

**Published:** 2022-05-05

**Authors:** Airi Tarutani, Tadashi Adachi, Hiroyasu Akatsu, Yoshio Hashizume, Kazuko Hasegawa, Yuko Saito, Andrew C. Robinson, David M. A. Mann, Mari Yoshida, Shigeo Murayama, Masato Hasegawa

**Affiliations:** 1 Department of Brain and Neuroscience, Tokyo Metropolitan Institute of Medical Science, 2-1-6 Kamikitazawa, Setagaya-ku, Tokyo, 156-8506 Japan; 2 Division of Neuropathology, Department of Brain and Neurosciences, Faculty of Medicine, Tottori University, Tottori, 683-8503 Japan; 3 Department of Neuropathology, Choju Medical Institute, Fukushimura Hospital, Aichi, 441-8124 Japan; 4 Department of Community-Based Medical Education, Nagoya City University Graduate School of Medical Sciences, Aichi, 467-8601 Japan; 5 Division of Neurology, National Hospital Organization, Sagamihara National Hospital, Kanagawa, 252-0392 Japan; 6 Department of Neuropathology, Tokyo Metropolitan Institute of Gerontology, Tokyo, 173-0015 Japan; 7 Department of Pathology and Laboratory Medicine, National Center Hospital, National Center of Neurology and Psychiatry, Tokyo, 187-8551 Japan; 8 Faculty of Biology, Medicine and Health, School of Biological Sciences, Division of Neuroscience and Experimental Psychology, Salford Royal Hospital, The University of Manchester, Salford, M6 8HD UK; 9 Department of Neuropathology, Institute for Medical Science of Aging, Aichi Medical University, Aichi, 480-1195 Japan; 10 Brain Bank for Neurodevelopmental, Neurological and Psychiatric Disorders, United Graduate School of Child Development, Osaka University, Osaka, 565-0871 Japan

**Keywords:** Tau, α-Synuclein, TDP-43, Tauopathy, Synucleinopathy, TDP-43 proteinopathy, Prion-like propagation, Strains

## Abstract

Intracellular accumulation of abnormal proteins with conformational changes is the defining neuropathological feature of neurodegenerative diseases. The pathogenic proteins that accumulate in patients' brains adopt an amyloid-like fibrous structure and exhibit various ultrastructural features. The biochemical analysis of pathogenic proteins in sarkosyl-insoluble fractions extracted from patients’ brains also shows disease-specific features. Intriguingly, these ultrastructural and biochemical features are common within the same disease group. These differences among the pathogenic proteins extracted from patients’ brains have important implications for definitive diagnosis of the disease, and also suggest the existence of pathogenic protein strains that contribute to the heterogeneity of pathogenesis in neurodegenerative diseases. Recent experimental evidence has shown that prion-like propagation of these pathogenic proteins from host cells to recipient cells underlies the onset and progression of neurodegenerative diseases. The reproduction of the pathological features that characterize each disease in cellular and animal models of prion-like propagation also implies that the structural differences in the pathogenic proteins are inherited in a prion-like manner. In this review, we summarize the ultrastructural and biochemical features of pathogenic proteins extracted from the brains of patients with neurodegenerative diseases that accumulate abnormal forms of tau, α-synuclein, and TDP-43, and we discuss how these disease-specific properties are maintained in the brain, based on recent experimental insights.

## Introduction

The pathological hallmark of neurodegenerative diseases is the accumulation of misfolded proteins in neurons and/or glial cells. The accumulation of amyloid-β (Aβ) and tau in the brains of patients with Alzheimer's disease (AD) was reported in the 1980s, and the group of diseases characterized by abnormal tau inclusions is referred to as tauopathies [46, 106, 315]. In 1997 and 1998, it was reported that α-synuclein (α-syn) is a major component of Lewy bodies (LBs) observed in the brains of patients with Parkinson's disease (PD), dementia with Lewy bodies (DLB) and glial cytoplasmic inclusions (GCIs) observed in multiple system atrophy (MSA) [26, 276, 302]. The group of diseases characterized by abnormal α-syn inclusions is termed synucleinopathies. In 2006, TAR DNA-binding protein of 43 kDa (TDP-43) was identified as a major component of the ubiquitin-positive inclusions that accumulate in the brains of patients with amyotrophic lateral sclerosis (ALS) and frontotemporal lobar degeneration (FTLD), and the diseases in which abnormal TDP-43 accumulates are collectively called TDP-43 proteinopathies [14, 216]. These abnormal proteins share common features such as insolubility in detergents, resistance to proteases and formation of amyloid-like fibrous structures, and exhibit prion-like properties, inducing the conversion of normal proteins into an abnormal form [26, 66, 128, 168, 217, 262, 274, 294, 304, 311, 312]. In addition, various post-translational modifications (PTMs), such as phosphorylation and ubiquitination, are detected in detergent-insoluble fractions extracted from patients’ brains [28, 156, 157]. Furthermore, histopathological studies of serial sections of post-mortem brains have shown that the intracerebral accumulation of these abnormal proteins correlates strongly with clinical symptoms and expands in a stereotypic manner [39, 40, 45, 145, 252, 253, 308]. Both the Braak and Murayama groups have proposed staging of the intracerebral expansion of these pathologies, which is widely used to evaluate disease progression [39, 40, 252, 253]. In AD, tau pathology first appears in the locus coeruleus and then expands from the hippocampus to the cerebral cortex through the cerebral limbic system [39, 42]. In argyrophilic grain dementia (AGD), tau pathology develops along the anterior and posterior medial temporal lobe from the ambient gyrus and further extends to the septum, insular cortex and anterior cingulate gyrus [253]. In PD, α-syn pathology spreads from the dorsal motor nucleus of the vagus nerve to the cerebral cortex along the pathway ascending the brainstem [40]. In DLB, the development of α-syn pathology has been reported to start at the olfactory bulb [252]. In ALS, TDP-43 pathology has been reported to spread from the motor cortex or brainstem to the temporal lobe [44, 45]. Furthermore, it has been reported that α-syn pathology was observed in the graft 10–24 years after transplantation of fetal dopamine neurons into PD patients [167, 183, 185]. These reports suggest the possibility that abnormal α-syn was released from the host cells and incorporated into the grafted cells. These pathological findings indicate that abnormal proteins accumulated in the brain self-amplify using themselves as a template, like prions, and spread from cell to cell along the neuronal circuit. This hypothesis is known as prion-like propagation, and self-templated amplification and cell-to-cell transmission of pathogenic tau, α-syn and TDP-43 have been demonstrated in in vitro and in vivo experimental models [66, 162, 199, 217, 236, 242, 314]. These experimental transmissions strongly support the idea that prion-like propagation is involved in the onset and progression of various neurodegenerative diseases. At present, the treatment of neurodegenerative diseases is limited to symptomatic therapy, but the development of novel disease-modifying therapies and agents targeting prion-like propagation is anticipated.

## Implications of ultrastructural and biochemical features for the diversity of prion strains

The prion hypothesis, which gave rise to the idea of prion-like propagation, was proposed as a mechanism to explain the pathogenesis of prion diseases by Prusiner [238]. Prion is a term that describes proteinaceous infectious particles that do not contain nucleic acids. Prion protein (PrP), which causes prion diseases such as Creutzfeldt–Jakob disease and bovine spongiform encephalopathy, is rich in α-helices in the normal state (PrP^C^), whereas when converted to the abnormal form (PrP^Sc^), it forms amyloids rich in β-sheet structures[171, 196, 222]. Then, PrP^Sc^ acts as a template and self-amplifies. Amyloid structures known as prion rods are observed in the detergent-insoluble fraction extracted from the brains of patients with prion diseases [32]. This conformational change enables PrP^Sc^ to acquire various properties different from those of PrP^C^. Biochemical analysis of infected animals and post-mortem tissues from patients revealed that PrP^Sc^ has unique features that are different from those of viruses, such as resistance to conventional virus inactivation methods including boiling and UV irradiation, insolubility in detergents, and resistance to proteases [206, 218, 239]. Immunoblot analysis of protease-treated PrP^Sc^ detects low-molecular-weight protease-resistant bands, and these banding patterns show diversity depending on the type of prion disease and the host species expressing PrP^C^ [139, 166, 224, 231]. Differences in sensitivity to proteases have also been reported [102, 137, 226]. What do these biochemical differences in PrP^Sc^ suggest? The diversity in the biochemical properties of PrP^Sc^ probably reflects distinct conformations of PrP^Sc^. In PrP “strains" with different conformations, the ultrastructural and biochemical features characterize the strain, and the structural information of PrP^Sc^ is crucial for self-amplification. Therefore, the structure of PrP^Sc^ may have significant implications for clinical symptoms and pathogenesis. PrP strains have been shown to have different infectivity and incubation periods in experimental animal models [248, 289]. It remains unclear what factors are involved in the formation of different strains from the same protein. However, some cofactors and PTMs may be involved [3, 52]. Recently, it has been demonstrated that strains are also present in amyloidogenic proteins that accumulate in the brains of patients with neurodegenerative diseases other than prion diseases. Among the pathogenic proteins associated with neurodegenerative diseases, tau, α-syn and TDP-43 are intracellularly accumulated and show common PTMs. In addition, there is evidence for a robust link between their accumulation and neurodegeneration [22, 114, 170, 200, 208, 307]. Therefore, we focus here on classification of the ultrastructural and biochemical features of neurodegenerative disease-specific strains of these three proteins.

## Ultrastructural and biochemical classification of pathogenic tau derived from human tauopathies

**Fig. 1:**
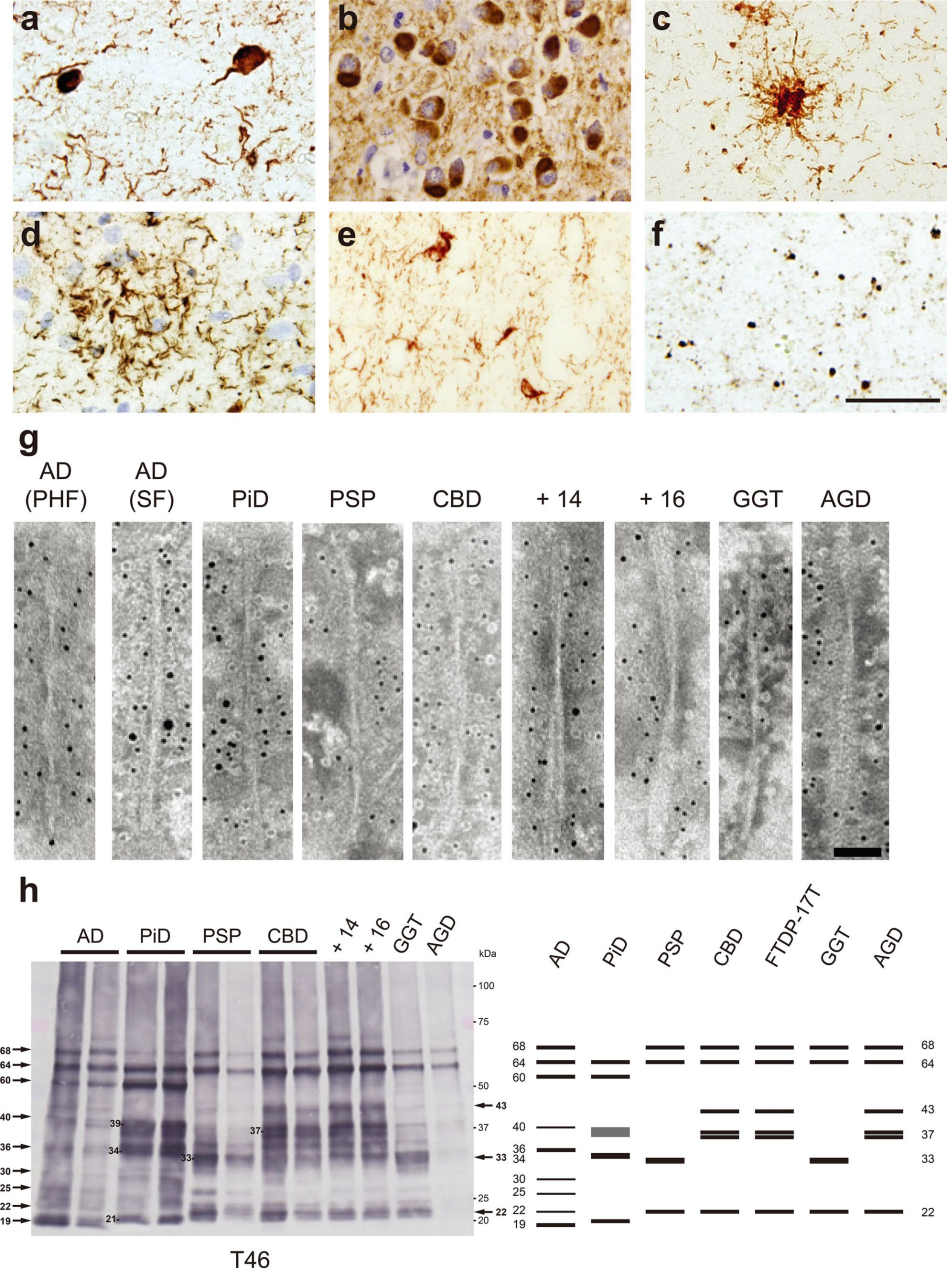
Ultrastructural and biochemical characterization of pathogenic tau extracted from human tauopathies. Immunohistochemistry of brain section from patients with tauopathies, stained with AT8 antibody. **a** Neurofibrillary tangles and neuropil threads in AD. **b** Pick bodies in PiD. **c** Tufted astrocyte in PSP. **d** Astrocytic plaque in CBD. **e** Neuronal inclusions and glial inclusions in FTDP-17T (+ 16). **f** Argyrophilic grains in AGD. Scale bar, 50 μm. **g** Immunoelectron microscopy of sarkosyl-insoluble fractions extracted from brains of tauopathy patients. Electron micrographs show fibrous structures positive for anti-tauC, after labeling with secondary antibody conjugated to 5 nm gold particles. Paired helical filaments (PHF, 10–20 nm in diameter) and straight filaments (SF, 15 nm in diameter) in AD, straight filaments (13–17 nm in diameter) in PiD, twisted ribbon-like filaments (15 nm in diameter) in PSP, twisted filaments (10–30 nm in diameter) in CBD, twisted filaments (7–25 nm in diameter) in FTDP-17T (+ 14, + 16), twisted ribbon-like filaments in GGT, and twisted filaments in AGD were observed. Scale bar, 50 nm. **h** Immunoblot analyses of sarkosyl-insoluble fractions prepared from brains of tauopathy patients. Sarkosyl-insoluble full-length tau (60, 64 and 68 kDa) and C-terminal fragments were detected with T46 antibody (residues 404–441). Disease-specific C-terminal fragments were also detected: 19, 22, 25, 30, 36 and 40 kDa bands in AD, 21, 34 and 39 kDa bands in PiD, 22 and 33 kDa bands in PSP and GGT, 22, 37 doublet and 43 kDa bands in CBD, FTDP-17T (+ 14, + 16) and AGD. All data are original for this review

Tau is a microtubule-associated protein localized mainly in axons and is one of the natively unfolded proteins [33, 47, 305]. In the adult human brain, tau exists in six isoforms consisting of 352–441 amino acids, resulting from alternative splicing of exon 2, exons 2 and 3, or exon 10 in the *MAPT* gene encoding tau [107]. The isoforms with 0, 1 or 2 inserts in the N-terminal repeat domains encoded by exons 2 and 3 are referred to as 0N, 1N and 2N, respectively. Furthermore, these tau isoforms are divided into three-repeat tau (3R tau) and four-repeat tau (4R tau), according to the number of repeats in the microtubule binding domain. The expression ratio of 3R tau to 4R tau is about 1:1 [109]. Under physiological conditions, tau functions to stabilize microtubules and promote their polymerization [67, 68, 141]. Tauopathies are classified into three groups: one in which 3R tau and 4R tau accumulate, one in which only 3R tau accumulates, and one in which only 4R tau accumulates. In AD and chronic traumatic encephalopathy (CTE), all six isoforms of human tau accumulate as neurofibrillary tangles (NFTs) and neuropil threads (Fig. 1a) [27, 113, 244]. In the very early stage of AD, tau-positive deposits with no obvious fibrous structure can be seen with a light microscope, and these are known as pre-tangles [27, 295]. However, electron microscopic studies have revealed fibrous structures in pre-tangles, suggesting that tau filaments in pre-tangles are not densely packed and unbundled, unlike NFTs [27, 288]. The occurrence of pre-tangles probably represents a preliminary stage of NFT formation. The Braak group has assessed pre-tangle stages with AT8-immunopositive tau, in addition to NFT stages with Gallyas silver staining [42]. Astrocytic tangles are also a neuropathological feature in CTE [203]. Primary age-related tauopathy (PART) is characterized by the absence of Aβ accumulation and by NFTs indistinguishable from those of AD [72]. In Pick’s disease (PiD), 3R tau accumulates in neurons as Pick's bodies, which are frequently located in interneurons in layer 2 of the cerebral cortex and the gyrus dentatus (Fig. 1b) [20, 232, 241]. In 4R tauopathies, 4R tau accumulates in neurons and glial cells in various pathological forms. Progressive supranuclear palsy (PSP) is characterized by tufted astrocytes, while corticobasal degeneration (CBD) is characterized by astrocytic plaques (Fig. 1c, d) [92, 134, 316]. In AGD, 4R tau accumulates as pre-tangles in neurons and argyrophilic grains (Fig. 1e) [38, 290]. Globular glial tauopathy (GGT) is pathologically characterized by globular tau-positive inclusions in glial cells [4, 169]. Although the majority of tauopathies are sporadic, more than 50 mutations in the exons and introns of the *MAPT* gene have been reported to be linked to the onset of tauopathies, and these cases are referred to as frontotemporal dementia and parkinsonism linked to chromosome 17 (FTDP-17T) [103, 143, 235, 275]. It has been reported that missense mutations on the *MAPT* gene alter the structure of normal tau and promote the formation of tau aggregates [110, 132]. In addition, mutations in the intron region of the *MAPT* gene affect alternative splicing and alter the expression ratio of 3R tau and 4R tau [120, 300]. These intronic mutations lead to the onset of tauopathies that present clinical symptoms and pathology similar to those of tauopathies in which tau isoforms accumulate (Fig. 1f) [65, 74, 143, 275]. Abnormal tau accumulated in the brain of patients with tauopathies is highly phosphorylated at numerous sites [121, 212]. These abnormal forms of tau lack the ability to bind to microtubules and show various PTMs, such as ubiquitination, acetylation, deamidation and oxidation, as well as phosphorylation [18, 157, 306]. The profiles of these PTMs in AD cases have been shown to change depending on the Braak stage [306]. First, in addition to the phosphorylation observed in the healthy brain, the number of sites and the frequency of phosphorylation in the proline-rich and C-terminal domains increase as the Braak stage progresses [306]. Furthermore, ubiquitination and acetylation in the microtubule-binding domain become more prominent at later Braak stages, suggesting that these PTMs may act as a degradation signal after NFTs are formed [306]. In addition to the PTMs common to tauopathies, disease-specific PTMs have been reported [157]. Non-phosphorylation of S356 and deamidation of N279 are distinctive features of insoluble tau extracted from AD cases [77, 157]. PiD is the only tauopathy in which S262 is not phosphorylated [16]. Multiple disease-specific ubiquitination (K343, K353, K369 and K375) is detected in CBD, whereas PSP is characterized by less ubiquitination [157]. Unlike other tauopathies, tau deposits in AGD cases have been reported to lack acetylation of K279 [119]. The abnormal tau is also known to be increased not only in the brain, but also in cerebrospinal fluid (CSF) and blood of patients with tauopathies [75, 151, 201, 299]. It has been reported that the influx of abnormal tau into CSF and blood may be a result of aging-related disruption of the blood–brain barrier, and that specific phosphorylation sites of tau in CSF and plasma may be useful as biomarkers to identify the early stage of tauopathies [149, 210, 278]. Neurofilament proteins and heparansulfate proteoglycans are known to show co-immunoreactivity in tau pathology, and neurofilament light chain is used as a biomarker of neurodegeneration [11, 220, 229, 271]. Heparansulfate proteoglycans have been reported to be involved in cell-to-cell transmission of pathogenic tau [140, 161].

In the brain of patients with tauopathies, abnormal tau accumulates as amyloid-like filaments rich in β-sheets. In 1963, the fibrous structure in the brain of AD patients was first reported on the basis of an electron microscopic study [163]. Furthermore, it has been revealed that this twisted fibrous structure is tau filaments composed of two protofilaments, known as paired helical filaments (PHFs), and that PHFs and straight filaments (SFs) are observed in the brains of AD patients [73, 112, 180]. Electron microscopy of the sarkosyl-insoluble fraction extracted from AD cases shows that PHFs are 10–20 nm in diameter and twisted with 80 nm periodicity, while SFs are 15 nm in diameter (Fig. 1g). In other tauopathies, abnormal tau also forms various disease-specific fibrous structures [19, 108, 284]. Tau filaments extracted from PiD cases are 13–17 nm in diameter and straight or twisted (Fig. 1g). Tau filaments 15 nm in diameter from PSP cases are loosely twisted ribbons, while those from CBD cases are 10–30 nm in diameter and twisted with 140 nm periodicity (Fig. 1g). In FTDP-17T (intron 10 mutations + 14 and + 16), tau filaments 7–25 nm in diameter are twisted with 240 nm periodicity, and these structures are different from those of filaments extracted from PSP and CBD cases (Fig. 1g). Tau filaments from GGT are ribbon-like, while those from AGD are twisted with a relatively long periodicity (Fig. 1g). In recent years, cryogenic electron microscopy (cryo-EM) analysis of sarkosyl-insoluble fractions extracted from the brains of patients with tauopathies has revealed the core structures of tau filaments that characterize each disease [266]. A C-shaped core structure consisting of V306-F378 in 3R tau and G304-E380 in 4R tau was identified in the brain of one individual with AD (Table 1) [98]. Although the protofilaments in PHFs and SFs share a common fold, the interfaces of the protofilaments are different (Table 1) [98]. The AD fold was also identified in insoluble fractions extracted from multiple AD cases, and the structures of tau filaments extracted from a familial AD case with a V717F mutation in the *APP* gene, which produces Aβ, were identical to those of sporadic AD cases [90]. Tau filament structures identical with those in sporadic AD cases have also been found in other tauopathies including PART, posterior cortical atrophy (PCA), familial British dementia (FBD) and familial Danish dementia (FDD) and in prion diseases including PrP cerebral amyloid angiopathy (PrP-CAA) and Gerstmann-Sträussler-Scheinker disease (GSS) [127, 265, 266]. In CTE cases, a fibrous core structure consisting of K274-R379 in 3R tau and S305-R379 in 4R tau was identified, and the CTE fold was different from the AD fold (Table 1) [91]. A nonproteinaceous molecule was also present within the CTE fold (Table 1) [91]. The J-shaped core structure of tau filaments extracted from PiD cases involves K254-F378 in 3R tau, and although most of the filaments were of single protofilament type, twisted filaments composed of two protofilaments were also identified (Table 1) [89]. Tau filaments extracted from CBD cases consist of single protofilament and doublet types, and a CBD fold consisting of K274-E380 in 4R tau, having a nonproteinaceous molecule within the fold, was identified (Table 1) [322]. It has been reported that the pre-tangles observed in CBD cases show a different fibrous morphology from those of AD cases, which may be due to differences in the stability and the interaction between the two protofilaments of AD and CBD filaments [21, 282, 288]. The PSP fold, which is structurally different from the CBD fold, consists of G272-N381 in 4R tau and is identical in typical and atypical PSP cases (Table 1) [266]. In GGT cases, a fibrous core structure consisting of G272-R379 in 4R tau, akin to the PSP fold, was identified, and while only protofilament-type tau filaments were found in PSP cases, multiple structures consisting of two protofilaments packed at different interfaces were found in type I and type II cases in the three types of GGT (Table 1) [266]. In AGD, FTDP-17T (intron 10 mutations + 3 and + 16) and aging-related tau astrogliopathy (ARTAG) cases, a fibrous core structure akin to the CBD fold, consisting of G273-D387 or N279-N381 in 4R tau, was identified (Table 1) [266]. Intriguingly, the disease-specific PTMs in the tauopathies described above are detected in and around these fibrous core structures [157]. The non-phosphorylation at K356 in AD cases and S262 in PiD cases is explained by the fact that these residues are in locations that are not accessible from the outside in each fold, suggesting that phosphorylation at these residues may be able to occur after the fold formation in tauopathies [89, 98]. These disease-specific tau fibrous core structures and the correspondence of these structures within the same disease group provide a direct demonstration of tau strain formation in the patient’s brain. Although the nonproteinaceous molecules within the CTE fold and the CBD fold have not been identified, these molecules may cause the structural differences from the AD fold and the AGD/FTDP-17T/ARTAG fold, respectively. Recombinant tau purified from *E. coli* also forms amyloid-like filaments akin to patient-derived tau filaments in the presence of polyanions such as heparin and dextran sulfate [111, 129]. Structural analysis of these synthetic tau filaments by cryo-EM revealed one type of fibrous core structure from synthetic 2N3R tau filaments and four types of fibrous core structure from synthetic 2N4R tau filaments [321]. However, these fibrous core structures are not consistent with any of those derived from patients’ brains. This difference may be attributed to the effect of polyanions, which are essential for the formation of synthetic tau filaments. On the other hand, it has been reported that a synthetic peptide consisting of tau I297-E391 forms PHF-like filaments without polyanions [5, 6]. It has also been suggested that some cell-derived factors and cofactors influence tau filament formation, and that the nonproteinaceous molecules in the CTE fold and the CBD fold may originate from the glial cell environment [97, 309].

**Table 1:**
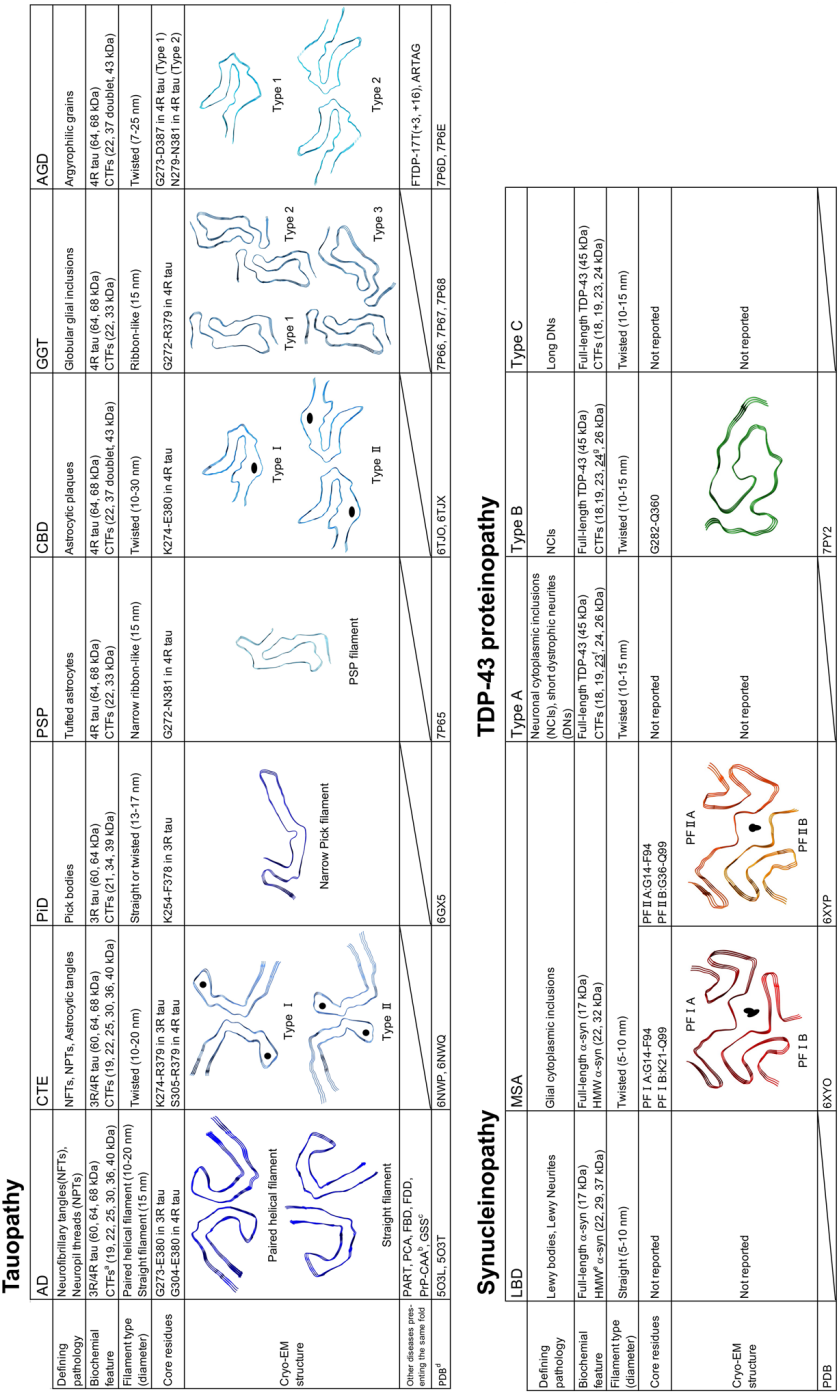
Summary of structural and biochemical features of patient-derived pathogenic tau, α-syn and TDP-43

^a^*CTF* C-terminal fragment

^b^PrP-CAA with mutation Q160X in the *PRNP* gene

^c^GSS with mutation F198S in the *PRNP* gene

^d^*PDB* protein data bank ID number

^e^*HMW* high-molecular-weight

^f^CTF at 23 kDa is the most intense band in Type A

^g^CTF at 24 kDa is the most intense band in Type B

Consistent with the structural diversity of tau filaments, the banding patterns of abnormal tau shown by immunoblotting of sarkosyl-insoluble fractions extracted from the brains of patients biochemically characterize tauopathies [15, 284]. Six isoforms of full-length tau are presented as 60, 64, and 68 kDa bands in AD cases, and three isoforms of full-length 3R tau are detected as 60 and 64 kDa bands in PiD cases (Fig. 1h). In 4R tauopathies, including PSP, CBD, FTDP-17T (+ 14 and + 16), GGT, and AGD cases, three isoforms of full-length 4R tau are detected as 64 and 68 kDa bands (Fig. 1h). Tau C-terminal fragments (CTFs) detected by antibodies that recognize the C-terminal of tau also characterize tauopathies. CTFs of 19, 22, 25, 30, 36, and 40 kDa are detected in AD cases, and 21, 34, and 39 kDa in PiD cases (Fig. 1h). CTFs resembling those in AD cases have been reported in CTE cases [219]. In PSP and CBD cases, different CTFs of 22, 33 kDa and 22, 37 doublet, 43 kDa, respectively, are detected (Fig. 1h). These CTF banding patterns are common within the same disease group. The CTF banding patterns of other 4R tauopathies can be divided into PSP-type and CBD-type. CTFs of insoluble tau extracted from GGT cases are PSP-type, whereas CTFs of insoluble tau extracted from FTDP-17T (+ 14, + 16) and AGD cases are CBD-type (Fig. 1h). These disease-specific CTF banding patterns may result from differences in the processing of full-length tau after filament formation, and also reflect the similarity of structural features of tau filaments, as demonstrated by the above cryo-EM structural analyses. The PSP and GGT folds show similar three-layered structures, whereas the CBD and AGD/FTDP-17T folds show similar four-layered structures (Table 1). The trypsin-treated insoluble tau derived from patients’ brains also displays trypsin-resistant bands specific to each tauopathy [284]. Mass analysis of these trypsin-treated insoluble tau revealed disease-specific trypsin-resistant core sequences [284]. These sequences match the sequences of the fibrous core structure revealed by cryo-EM analysis.

## Experimental prion-like amplification and transmission of patient-derived tau strains

Insoluble tau extracted from a patient’s brain acts as seeds in vitro, in cultured cells, in primary cultures, and in animal brains, and induces seed-dependent tau aggregation. In in vitro systems such as protein misfolding cyclic amplification (PMCA) and real-time quaking-induced conversion (RT-QuIC), recombinant tau purified from *E. coli* and samples derived from patients with tauopathies are mixed and incubated with thioflavin, a ligand that specifically binds to β-sheet structure, with shaking or sonication [24, 59]. Seed-dependent tau aggregation is monitored in terms of thioflavin fluorescence intensity. It has been reported that RT-QuIC using brain homogenates can distinguish AD and CTE cases from PiD cases based on the reactivity with thioflavin T [172, 249]. Similarly, brain homogenates and CSFs from PSP and CBD cases can be diagnosed with RT-QuIC [250]. In addition, introduction of patient-derived tau seeds into cultured cells or primary cultures expressing tau induces intracellular tau aggregation [213, 287, 313]. Insoluble tau extracted from AD cases (AD-tau) recruits both 3R tau and 4R tau for aggregation, whereas insoluble tau extracted from PiD cases (PiD-tau) or 4R tauopathy cases causes aggregation of only 3R tau or 4R tau, respectively. Furthermore, it has been reported that the introduction of patient-derived tau seeds into fluorescence resonance energy transfer biosensor cells induces the formation of tau aggregates with various morphologies, and that these morphological differences are inherited during the passaging of cells [162]. Inoculation of patient-derived tau seeds into mouse brain also leads to the formation of tau pathology associated with the inoculated sample. Brain homogenates derived from AD, PSP, CBD and AGD cases induced inoculated-sample-associated tau pathology in human tau transgenic (Tg) mouse brain, whereas the brain homogenates derived from PiD cases did not induce Pick body-like tau pathology [66]. Tau pathologies associated with inoculated samples, including the cell type specificity, are also observed after the inoculation of wild-type mice with brain homogenates or insoluble fractions extracted from tauopathy cases [125, 213]. Furthermore, it has been reported that disease-associated tau pathology is formed in mice expressing all six isoforms of human tau [136]. AD-tau induced tau pathology involving all the tau isoforms, while PiD-tau and insoluble tau extracted from PSP cases (PSP-tau) and CBD cases (CBD-tau) induced tau pathology composed only of 3R tau or 4R tau, respectively [136]. These results suggest that the conformation of tau filaments accumulated in the brains of patients with tauopathies is involved in the formation of the tau pathologies that characterize each disease.

## Ultrastructural and biochemical classification of pathogenic α-synuclein derived from human synucleinopathies

**Fig. 2:**
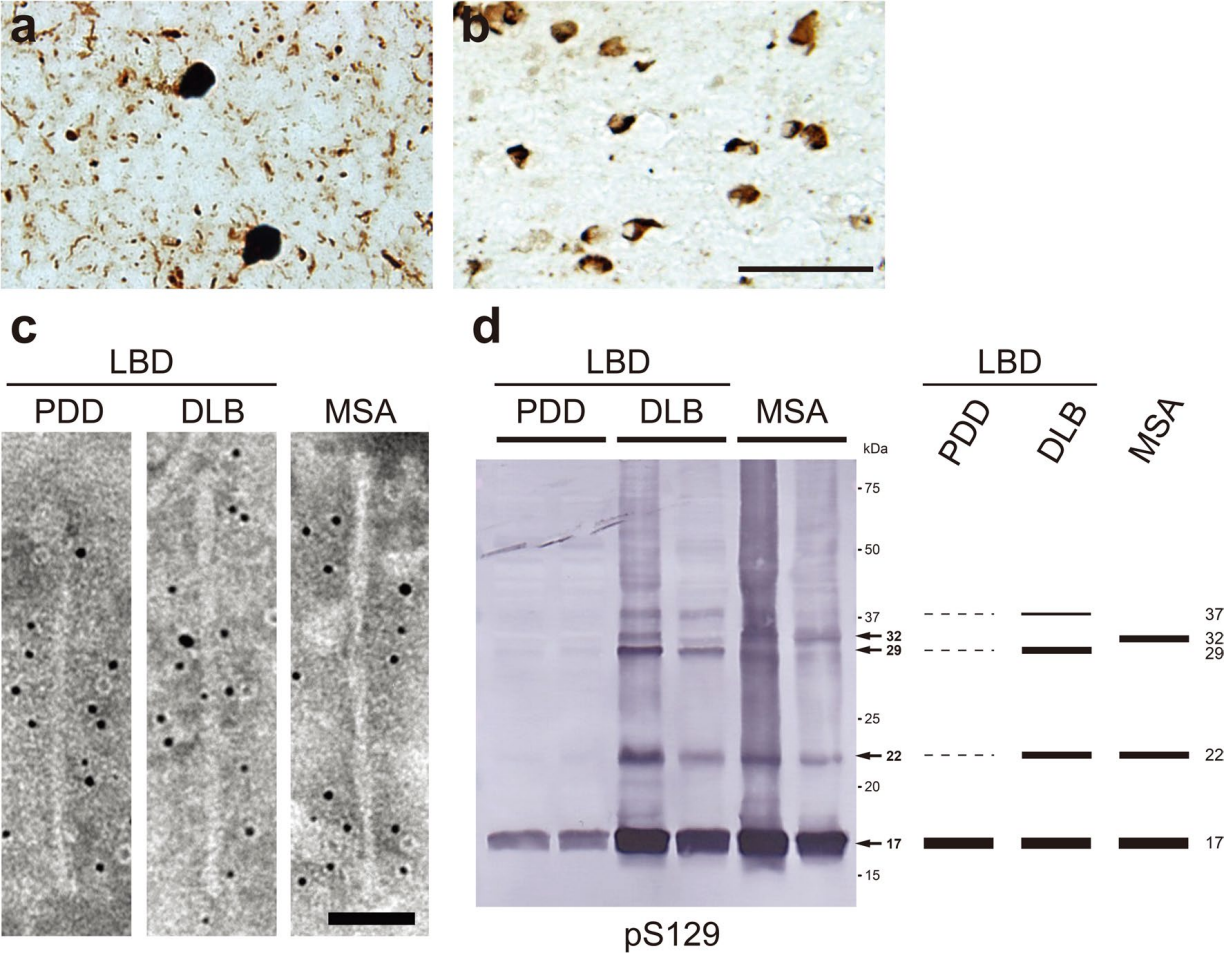
Ultrastructural and biochemical characterization of pathogenic α-syn extracted from human synucleinopathies. Immunohistochemistry of brain section from patients with synucleinopathies, stained with pS129 antibody. **a** Lewy bodies and Lewy neurites in DLB. **b** Glial cytoplasmic inclusions in MSA. Scale bar, 50 μm. **c** Immunoelectron microscopy of sarkosyl-insoluble fractions extracted from brains of synucleinopathy patients. Electron micrographs show fibrous structures with 5–10 nm in diameter positive for pS129, after labeling with secondary antibody conjugated to 5 nm gold particles. Straight filaments in LBD (PDD and DLB), and twisted filaments with 80–100 nm periodicity in MSA were observed. Scale bar, 50 nm. **d** Immunoblot analyses of sarkosyl-insoluble fractions prepared from brains of synucleinopathy patients. Sarkosyl-insoluble phosphorylated α-syn (17 kDa) was detected with pS129 antibody. Disease-specific high-molecular-weight α-syn species were also observed: 22, 29, and 37 kDa bands in LBD (PDD and DLB), and 22 and 32 kDa bands in MSA. All data are original for this review

α-Synuclein is a synaptic protein that is abundant in the brain and localizes to presynaptic terminals [148, 198]. It consists of 140 amino acids in three domains, the N-terminal domain, the hydrophobic region known as the NAC (non-amyloid core), and the C-terminal proline-rich hydrophobic region [296, 318]. Under physiological conditions, α-syn is a water-soluble, natively unfolded protein, and its function remains unclear [194]. However, the phenotype of α-syn-knockout mice has led to the suggestion that α-syn may be involved in the release of dopamine [1, 61]. It has also been shown that α-syn is involved in the regulation of SNARE complex formation, suggesting that it may act as a molecular chaperone [53, 62]. Synucleinopathies are classified into two major groups, Lewy body disease (LBD), which is characterized by α-syn inclusions in neurons, and MSA, which is characterized by α-syn inclusions mainly in oligodendrocytes. In LBD including PD, Parkinson's disease with dementia (PDD), and DLB, α-syn accumulates in neuronal cell bodies and neurites as LBs and Lewy neurites (LNs) (Fig. 2a) [276]. Diffuse α-syn-immunopositive deposits and pale bodies with fibrous structures are observed in neuronal cells of LBD cases, suggesting that they are formed in the preliminary stages of LB formation [76, 115, 301]. In addition to the Braak staging, the McKeith type is also widely used as a neuropathological staging system of LBD [40, 205]. Braak staging assesses the expansion of Lewy pathology, whereas the McKeith type classifies LBD into five neuropathological subtypes according to the severity of LB pathology in the anatomical regions [40, 204]. MSA is categorized into MSA with predominant parkinsonian features (MSA-P) and MSA with predominant cerebellar ataxia (MSA-C), and in both cases, α-syn accumulates in oligodendrocytes as GCIs (Fig. 2b) [105, 223, 302]. Abnormal α-syn accumulated in the brain of patients with synucleinopathies is phosphorylated at S129 and also partially ubiquitinated [9, 101, 130]. More than 90% of α-syn is phosphorylated in DLB patients’ brains, whereas only 4% is phosphorylated in healthy brains [101]. In LBD patients, phosphorylated α-syn pathology is detected not only in the brain, but also in peripheral tissues such as esophagus, heart, skin, adrenal gland, gut, and sympathetic ganglion [30, 283]. α-Syn is expressed in various peripheral tissues, and the spreading of α-syn pathology from peripheral tissues to the central nervous system has also been proposed [41, 135, 296]. In addition, it has been reported that the concentration of α-syn is increased in CSF and blood derived from patients with synucleinopathies, and this finding is expected to lead to a practical method for early diagnosis [34, 85, 257]. However, since α-syn is abundantly present in platelets, diagnosis using blood samples would need to be performed carefully [133]. Nine missense mutations (A30P, A30G, E46K, H50Q, G51D, A53T, A53E, A53V and E83Q) in the *SNCA* gene encoding α-syn have been reported to cause familial synucleinopathies [13, 159, 173, 182, 225, 233, 237, 319, 320]. These mutations have been experimentally found to exhibit differences in cytotoxicity, association with cell membranes and lipids, and filament formation as compared with wild-type [64, 84, 104, 153, 209, 247, 255]. Duplication and triplication of the *SNCA* gene also cause early-onset LBD [63, 94, 144, 268]. It has been suggested that PrP^C^ and lymphocyte-activation gene 3 (LAG3) bind to α-syn filaments and participate in cell-to-cell transmission of pathogenic α-syn as a receptor, but this remains controversial, with several reports suggesting non-binding of PrP^C^ to α-syn and lack of LAG3 expression in human neurons [71, 87, 175, 197, 297].

In the brains of patients with synucleinopathies, abnormal α-syn accumulates in the form of amyloid-like fibrous structures [273, 274, 286]. Fibrous structures in LBD brains were first reported in the 1960s [83, 246]. Electron microscopy of the sarkosyl-insoluble fraction extracted from the brains of patients with synucleinopathies shows α-syn filaments 5–10 nm in diameter, and there is a structural difference between α-syn filaments derived from LBD and MSA cases (Fig. 2c). α-Syn filaments extracted from LBD cases are straight, whereas those extracted from MSA cases are twisted with 80–100 nm periodicity (Fig. 2c). In addition, α-syn filaments derived from LBD cases are thinner than α-syn filaments derived from MSA cases (Fig. 2c). The core structures of α-syn filaments from MSA cases, including both MSA-C and MSA-P, have been identified by cryo-EM analysis [258]. Two types of α-syn filaments, type I and type II, have been identified [258]. Type I filaments comprise two protofilaments, PF-IA consisting of G14-F94 and PF-IB consisting of K21-Q99, while type II filaments comprise two protofilaments, PF-IIA consisting of G14-F94 and PF-IIB consisting of G36-Q99 (Table 1) [258]. Furthermore, additional densities between the two protofilaments, not due to α-syn, was seen in both type I and type II (Table 1) [258]. Although the details of these putative nonproteinaceous molecules remains unclear, it is suggested that they are involved in the assembly of the two protofilaments. Two-dimensional analysis showed that α-syn filaments extracted from DLB cases are not twisted, unlike those extracted from MSA cases [258]. The core structures of wild-type and familial synucleinopathy-related mutant-type synthetic α-syn filaments have also been reported [36, 37, 122, 186, 279, 280, 323]. As in the case of synthetic tau filaments, none of the structures of synthetic α-syn filaments are consistent with the structures of type I and type II filaments identified from MSA cases.

Abnormal α-syn accumulated in the brains of patients with synucleinopathies is detected by immunoblotting as a 17 kDa band (Fig. 2d) [286]. The high-molecular-weight bands detected by α-syn antibodies are different between LBD and MSA: 22, 29, and 37 kDa bands are detected in LBD, while 22 and 32 kDa bands are detected in MSA (Fig. 2d) [286]. It has been reported that each of the three high-molecular-weight bands observed in DLB is ubiquitinated α-syn, suggesting that the PTM patterns of abnormal α-syn are different in LBD and MSA [9]. The sensitivity to protease and the protease-resistant banding patterns of insoluble α-syn extracted from LBD and MSA cases are also different [228]. Insoluble α-syn extracted from MSA cases (MSA-syn) is more resistant to protease K and pronase than insoluble α-syn from LBD cases (LBD-syn) [228]. Furthermore, MSA-syn has high SDS solubility, while LBD-syn shows low SDS solubility [55].

## Experimental prion-like amplification and transmission of patient-derived α-synuclein strains

Like tau, insoluble α-syn extracted from the brain of patients with synucleinopathies exhibits prion-like properties and causes seed-dependent α-syn aggregation in vitro and in vivo. Heterogeneity of the prion-like seeding activity has also been reported, depending on the α-syn strain [228, 286, 314]. Similar to the case of tauopathy, abnormal α-syn in brain, CSF and skin samples derived from patients with synucleinopathies have been shown to be specifically detected by PMCA and RT-QuIC methods and to be useful biomarkers in the early stages of synucleinopathies [88, 174, 245, 256, 264]. PMCA analysis using brain samples and CSF derived from synucleinopathy cases has revealed differences in the seeding activity of α-syn derived from PD cases (PD-syn) and MSA-syn [263]. MSA-syn induced a rapid increase in thioflavin fluorescence intensity compared to PD-syn, while the fluorescence value at the plateau when PD-syn was added was much higher than when MSA-syn was added [263]. The variation in fluorescence values at the plateau may reflect the structural difference between PD-syn and MSA-syn in terms of the binding mode of α-syn filaments to thioflavin. The α-syn aggregates amplified by PMCA from PD cases showed four bands of 4–10 kDa after protease K treatment, whereas only two bands of 4 and 6 kDa were detected in the case of α-syn aggregates derived from MSA cases [263]. These two types of protease K-resistant banding patterns suggest that distinct PMCA products are formed depending on the template. The difference in seeding activity between LBD-syn and MSA-syn (i.e., MSA-syn induces greater intracellular α-syn aggregation than LBD-syn) is also observed in cultured cells and primary cultures [228, 286, 314, 317]. Furthermore, the potent prion-like character of MSA-syn is manifested in the brains of α-syn A53T Tg mice and wild-type mice [228, 240, 286, 304]. However, in mouse brains inoculated with MSA-syn, α-syn pathology is observed in neurons, but not in oligodendrocytes [178, 286, 304]. This may be explained by the level of α-syn expression in oligodendrocytes. In situ hybridization analyses of healthy brains and MSA brains indicate that α-syn expression is very low in oligodendrocytes [152, 207, 272]. It is possible that normal or oligomeric α-syn before the formation of β-sheet structure is secreted from neurons and incorporated into oligodendrocytes. α-Syn pathology in oligodendrocytes has been shown to form in Tg mice that express α-syn in oligodendrocytes [228]. Further studies are required to clarify the involvement of neuron-derived α-syn in the GCI formation in MSA. In addition, the cellular environment of oligodendrocytes, which is different from that of neurons, may affect the formation of α-syn filaments, resulting in the generation of α-syn strains. The possibility that different cellular environments may be involved in the formation of distinct α-syn strains has been suggested based on studies using recombinant α-syn. The synthetic α-syn filaments formed under various physiological conditions exhibit distinct morphological and physical properties, and exhibit different seeding activity, cytotoxicity and proteosome activity in vitro, in cultured cells and in primary cultures [35, 118, 281]. Synthetic α-syn filaments formed in the presence of physiological salt concentration showed significant cytotoxicity, while those formed in the absence of salt showed high seeding activity in rat brain [227]. Intracerebral inoculation of these synthetic α-syn strains also causes various α-syn pathological morphologies and propagation patterns in the brains of α-syn A53T Tg mice and wild-type mice [177, 281].

## Ultrastructural and biochemical classification of pathogenic TDP-43 derived from human TDP-43 proteinopathies

**Fig. 3:**
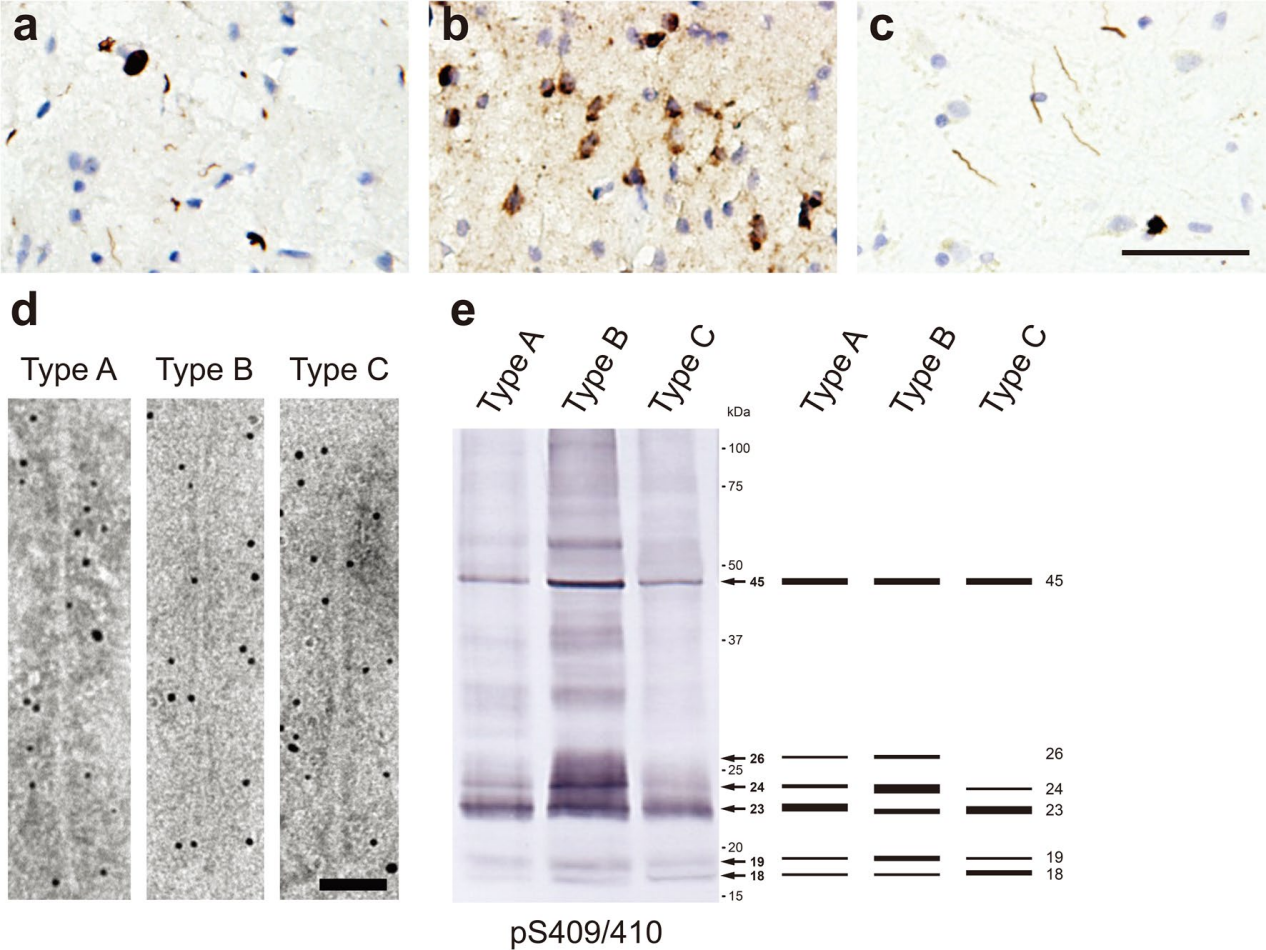
Ultrastructural and biochemical characterization of pathogenic TDP-43 extracted from human TDP-43 proteinopathies. Immunohistochemistry of brain section from patients with TDP-43 proteinopathies, stained with pS409/pS410 antibody. **a** Neuronal cytoplasmic inclusions (NCIs) and short degenerative neurites (DNs) in FTLD-TDP Type A. **b** NCIs in FTLD-TDP Type B. **c** Long DNs in FTLD-TDP Type C. Scale bar, 50 μm. **d** Immunoelectron microscopy of sarkosyl-insoluble fractions extracted from FTLD-TDP (type A, type B, type C) patients. Electron micrographs show fibrous structures with 10–15 nm in diameter positive for pS409/pS410, after labeling with secondary antibody conjugated to 5 nm gold particles. Scale bar, 50 nm. **e** Immunoblot analyses of sarkosyl-insoluble fractions prepared from brains of patients withTDP-43 proteinopathies. Sarkosyl-insoluble phosphorylated TDP-43 (45 kDa) was detected with pS409/410 antibody. Subtype-specific C-terminal fragments were also observed: 18, 19, 23, 24 and 26 kDa bands in type A, 18, 19, 23, 24 and 26 kDa bands in type B, 18, 19, 23 and 24 kDa bands in type C. All data are original for this review

TDP-43 is an RNA-binding nuclear protein localized mainly in the nucleus [25]. It consists of 411 amino acids with a nuclear translocation signal (NLS) sequence, and is composed of the N-terminal domain, two RNA recognition motif domains, and the C-terminal glycine-rich domain [48, 221]. The physiological functions of TDP-43 include the regulation of processing functions such as pre-mRNA splicing, and it is involved in the transport of mRNA from the nucleus to the cytoplasm [7, 48, 49, 69, 291]. TDP-43 was reported to be a major component of the ubiquitin-positive inclusion bodies observed in the brains of patients with FTLD and ALS [14, 216]. The abnormal TDP-43 inclusions mostly accumulate in the cytoplasm, and the nuclear localization of TDP-43 and its physiological functions are lost [50, 310]. FTLD-TDP, in which phosphorylated and ubiquitinated TDP-43 accumulates, is classified into five subtypes (type A-E) based on differences in pathology and distribution [179, 193]. Type A is defined by neuronal cytoplasmic inclusions (NCIs) and short dystrophic neurites (DNs) (Fig. 3a) [193]. Type B, which includes ALS and FTLD with motor neuron disease, presents predominantly NCI pathology (Fig. 3b) [193]. Type C is characterized by long DNs (Fig. 3c) [193]. Type D presents predominantly intranuclear inclusion pathology [192, 193]. Type E is characterized by granulofilamentous neuronal inclusions and very fine, dot-like neuropil inclusions [179]. TDP-43-immunopositive diffuse or granular or dash-like cytoplasmic inclusions are observed in the brains of FTLD-TDP patients, and the formation of these pre-inclusions may represent the early stage of pathogenesis in TDP-43 proteinopathy [43, 78, 211]. Limbic-predominant age-related TDP-43 encephalopathy (LATE) is neuropathologically characterized by the accumulation of phosphorylated TDP-43 in the limbic system and leads to AD-like symptoms in elderly individuals [214]. Missense mutations on the *TARDBP* gene encoding TDP-43 are associated with the onset of ALS and FTLD-TDP [160]. More than 30 mutations have been reported, and most of them are located in the C-terminal low-complexity domain [155, 277]. Mutations in other RNA-binding proteins, including hnRNPA1, hnPA2B1 and MATR3, and a hexanucleotide repeat expansion in the *C9ORF72* gene also cause TDP-43 proteinopathy, suggesting that the interaction of these RNA-binding proteins with TDP-43 and disruption of RNA metabolic homeostasis are involved in the intracytoplasmic accumulation of TDP-43 [80, 154, 164, 243]. Recently, the association between the formation of stress granules and the accumulation of TDP-43 in the cytoplasm has been investigated [10, 81]. Under various stress conditions, such as oxidative stress and heat shock, stress granules, which are membrane-less organelles composed of RNA and RNA-binding proteins, are formed via liquid–liquid phase separation, and it has been reported that they take up endogenous TDP-43 [70, 95, 187]. However, it is not clear whether stress granule formation directly leads to TDP-43 pathogenesis, and further studies are required.

Immuno-electron microscopic studies of brain sections from cases of ALS and FTLD-with ubiquitin-positive inclusions have shown the presence of TDP-43 and ubiquitin-positive bundles of straight filaments 10–20 nm in diameter in neurons [147, 165, 188]. Amyloid-like filaments 10–15 nm in diameter are also observed by electron microscopy of insoluble fractions extracted from the brains of patients with TDP-43 proteinopathy (Fig. 3d) [128, 217]. The ultrastructural features of TDP-43 filaments derived from types A-C are distinct for each subtype, and TDP-43 filaments derived from type B are thinner than those derived from other subtypes (Fig. 3d). Synthetic peptides G274-F313 and G314-N353 of TDP-43 were reported to form filaments in vitro, and these filaments possess seeding activity that recruits wild-type TDP-43 or TDP-43 lacking NLS expressed in cultured cells for aggregation, suggesting that residues 274–353 are important for TDP-43 filament formation [267]. Furthermore, cryo-EM analysis of TDP-43 filaments formed from SegA (residues 311–360) and SegB (residues 286–331 including the ALS hereditary mutation A315E) peptides has revealed three different dagger-shaped core structures from SegA filaments and one core structure composed of four R-shaped folds from SegB filaments [56]. In addition, a fibrous core structure consisting of F276-M414 has been identified from TDP-43 filaments composed of the low-complexity domain (residues 267–414) formed under a mildly acidic condition (pH 4) [184]. Cryo-EM analysis of TDP-43 filaments extracted from two individuals diagnosed as ALS with FTLD identified a double-spiral-shaped fold consisting of G282-Q360, which is different from the fibrous core structures of the three synthetic TDP-43 filaments described above (Table 1) [23]. The structure of TDP-43 filaments extracted from brains of patients with other subtypes of FTLD-TDP should also be amenable to cryo-EM analysis. Immunoblotting with phosphorylated S409/S410 antibody of sarkosyl-insoluble fractions extracted from the brains of patients with TDP-43 proteinopathy shows a 45 kDa band of phosphorylated full-length TDP-43 and subtype-specific CTFs bands (Fig. 3e) [128, 131]. In type A, major bands of 23, 24, and 26 kDa and minor bands of 18 and 19 kDa are present and the 23 kDa band is the most intense (Fig. 3e). Similarly, CTFs of 18, 19, 23, 24, and 26 kDa, with the most intense band at 24 kDa, are seen in type B (Fig. 3e). In type C, major bands of 23 and 24 kDa, of which the 23 kDa band is more intense, and minor bands of 18 and 19 kDa are seen (Fig. 3e). These banding patterns of CTFs are common within the same subtype group and may reflect differences in the N-terminal truncation of full-length TDP-43 among the subtypes [294]. A single CTF banding pattern was detected even in the insoluble fractions extracted from different brain regions and spinal cord of one individual [294].It has been reported that the protease-resistant banding patterns of patient-derived insoluble TDP-43 are also common within the same subtype group, but are different between subtypes [294]. In addition, the major PTMs detected in insoluble fractions extracted from ALS cases are phosphorylation, oxidation and deamidation, and partial ubiquitination is also detected [156]. These PTMs are mainly located in the C-terminal domain [156]. While phosphorylation at S403/S404 and at S409/S410 is a definitive pathological marker for all subtypes, differences in immunoreactivity to phosphorylation at S369 have been reported: TDP-43 pathology in type B and type C is pS369-positive, whereas type A is pS369-negative [128, 215]. As with tau and α-syn, these ultrastructural and biochemical variations may reflect differences in the conformation of abnormal TDP-43 accumulated in the brain, suggesting the formation of TDP-43 strains in TDP-43 proteinopathies. The ultrastructural and biochemical features of pathogenic TDP-43 that accumulate in other subtypes and LATE require further investigation.

## Experimental prion-like amplification and transmission of patient-derived TDP-43 strains

Patient-derived insoluble TDP-43 exhibits prion-like properties in vitro and in vivo. An RT-QuIC study found that abnormal TDP-43 in brain homogenates and CSF derived from ALS and FTLD cases were amplified using full-length TDP-43 and truncated TDP-43 (residues 263–414) purified from *E. coli* as substrates [259]. Abnormal TDP-43 could be used as a biomarker for early diagnosis. Introduction of insoluble TDP-43 extracted from the brains of patients with FTLD-TDP types A-C into cultured cells expressing wild-type TDP-43 or TDP-43 lacking NLS causes seed-dependent accumulation of phosphorylated TDP-43 [217]. Furthermore, the insoluble fractions extracted from the cells into which patient-derived TDP-43 seeds had been introduced showed CTF banding patterns similar to those of the patient-derived original seeds [217]. Insoluble TDP-43 extracted from FTLD-TDP type A-C cases by Sarkospin also shows differences of cytotoxicity and seeding activity in cultured cells depending on the subtype [79, 176]. In addition, it has been reported that insoluble TDP-43 extracted from ALS cases induces the formation of phosphorylated TDP-43 inclusions with various morphologies, as observed in the brain of ALS patients in cultured cells expressing wild-type TDP-43, and that the prion-like property of insoluble TDP-43 accumulated in cultured cells is inherited after passaging of the cells [269]. In vivo, intracerebral inoculation of the insoluble fractions extracted from the brains of patients with sporadic FTLD-TDP and familial FTLD-TDP with the *GRN* or *C9ORF72* gene mutations into Tg mice expressing human TDP-43 in the cytoplasm reproduced TDP-43 pathology associated with the inoculated samples in a time-dependent manner [236]. Inoculation of these patient-derived samples into wild-type mice also induced TDP-43 pathology, although the pathology was not as abundant as in the case of inoculation into Tg mice [236].

## How are the characteristic structural and biochemical properties of each disease maintained in the patient's brain?

As we have described, abnormal proteins that accumulate in the brains of patients exhibit ultrastructural and biochemical properties that are characteristic of each disease. It is worth emphasizing that these properties are common in the same disease group, and even in different brain regions of one individual. These results suggest that pathogenic tau, α-syn and TDP-43 spread throughout the brain, retaining their disease-specific structural and biochemical properties.

**Fig. 4:**
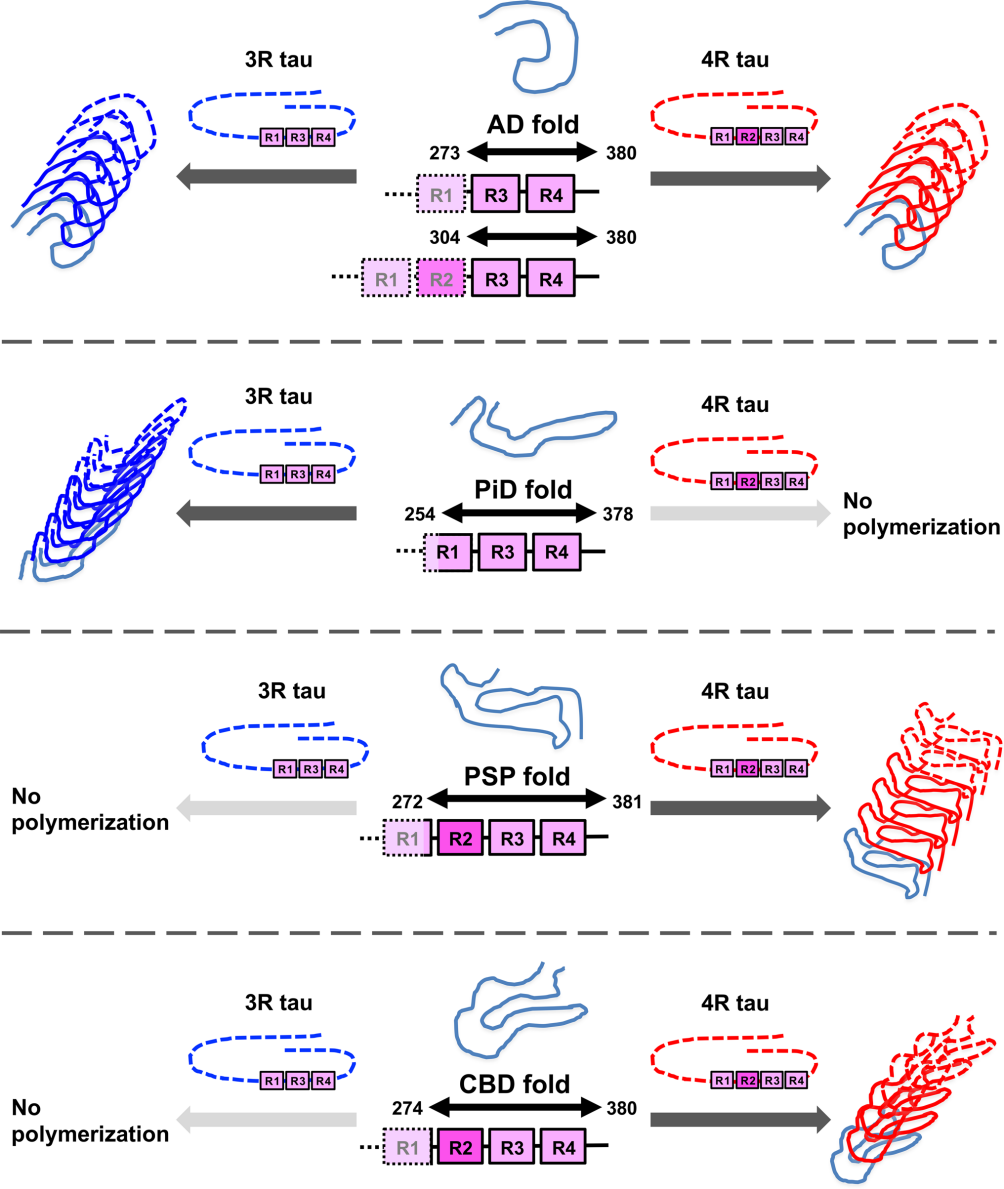
Full matching of amino acid sequence between template and substrate is essential for template-dependent amplification of tau filaments. Fibrous core regions of tau filaments extracted from brains of AD, PiD, PSP and CBD patients identified by cryogenic electron microscopic studies explain the templated tau amplification and tau filament formation observed in our cellular model. The core region of AD-tau (G273-E380 in 3R tau and G304-E380 in 4R tau), which recruits both 3R tau and 4R tau for seeded aggregation, consists of amino acid sequences common to 3R tau and 4R tau. On the other hand, PiD-tau, PSP-tau and CBD-tau consist of 3R tau or 4R tau specific amino acid sequences: K254-F378 in 3R tau, G272-N381 in 4R tau and K274-E380 in 4R tau. Therefore, PiD-tau, PSP-tau and CBD-tau recruit only tau substrates that match the template for seeded aggregation, and do not induce aggregation when the template and substrate are mismatched. The amplification and intracerebral expansion of tau filaments with the same structure by this mechanism leads to the pathological diversity of tauopathies

To further explore this possibility, we examined whether disease-specific tau aggregation and filament formation were reproduced in cultured cells [287]. Insoluble tau extracted from the brains of patients with tauopathies was introduced into SH-SY5Y cells expressing full-length 3R tau or 4R tau. PiD-tau induced only 3R tau aggregation, while PSP-tau and CBD-tau induced only 4R tau aggregation. AD-tau recruited both 3R tau and 4R tau for aggregation. This strain-specific tau aggregation was also observed in cells expressing both 3R tau and 4R tau. In addition, immunoelectron microscopy of insoluble tau accumulated in SH-SY5Y cells showed abundant tau filaments labeled by antibody to the tag protein expressed in the cells, and their structures resembled those of filaments derived from the patients’ brains. This template-dependent amplification of tau filaments observed in cultured cells can be explained in terms of the core structure of tau filaments extracted from the brain. Since the AD fold is composed of the common part of 3R tau and 4R tau, AD-tau can recruit both 3R tau and 4R tau for seed-dependent aggregation (Fig. 4). On the other hand, the PiD fold, the PSP fold and the CBD fold contain 3R tau- or 4R tau-specific sequences and therefore, polymerization of β-sheet structure is not promoted when there is a mismatch between template and substrate (Fig. 4). In other words, the structure of the tau filaments determines which tau isoform is recruited for seed-dependent aggregation, and is the determinant of the formation and spreading of tau pathology in the brain. Trypsin-treated patient-derived tau seeds, which were digested outside the tau fibrous core region (fuzzy coat), caused the same seed-dependent aggregation as untreated tau seeds, supporting the idea that the fibrous core region plays a cardinal role in the amplification of tau filaments. Thus, our results suggest that the template-dependent amplification of pathognomonic proteins in the brain leads to the pathological diversity in the same proteinopathy.

**Fig. 5:**
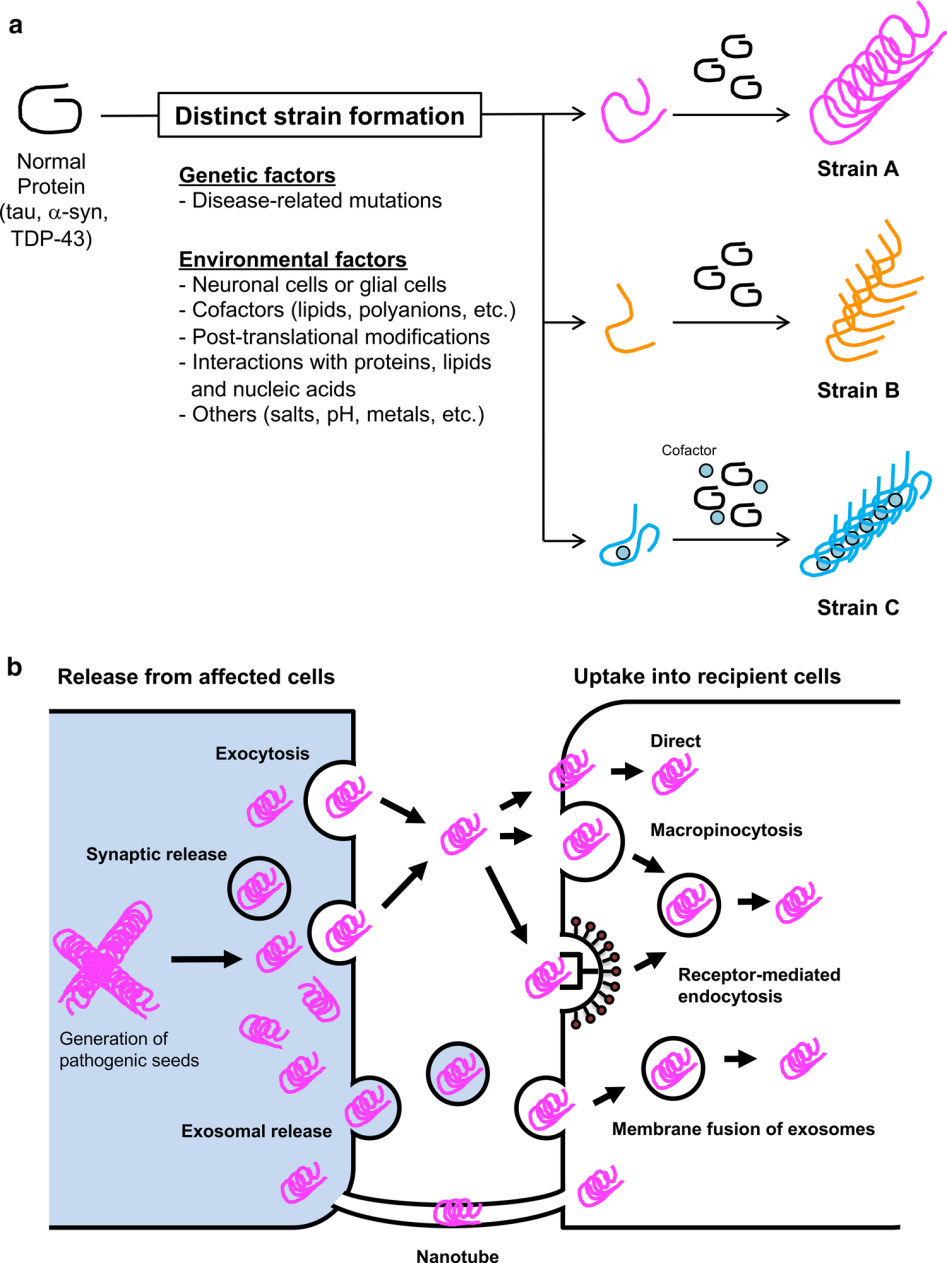
Strain formation and cell-to-cell transmission in neurodegenerative diseases. **a** Prion-like proteins, including tau, α-syn and TDP-43, can adopt various misfolded forms. The variety of misfolding is caused by genetic and/or environmental factors, resulting in the formation of strains with distinct conformations. Disease-associated mutations alter the core structure of filaments and the interaction between two protofilaments. Differences in the cellular environment between neuronal and glial cells may also contribute to the various types of misfolding. The interaction of prion-like proteins with cell type-specific co-factors during misfolding would lead to the formation of disease-specific filaments. Post-translational modifications, interactions with other proteins, lipids and nucleic acids, as well as differences in salt concentration, pH and metals, may also be involved in the formation of distinct strains. **b** Pathogenic proteins amplified and accumulated in cells have proposed to transmit as seeds from cell to cell and then spread throughout the brain. Possible mechanisms of cell-to-cell transmission include extracellular release of pathogenic seeds via exocytosis or in synaptic vesicles or exosomes, followed by incorporation into neighboring cells either directly or via macropinocytosis or receptor-mediated endocytosis. Alternatively pathogenic seeds may be taken up into cells by cell membrane fusion of exosomes containing seeds. Cell-to-cell transmission of pathogenic seeds via tunneling nanotubes has also been suggested

The requirement of amino acid sequence identity between template and substrate for template-dependent amplification is also clearly demonstrated by the in vitro and in vivo observation of low seeding and propagation efficiency caused by species barriers [191, 251]. Seven residues in 140 amino acids differ between the amino acid sequences of human and mouse α-syn, and it has been reported that the seeding activity is significantly decreased when the substrate and template are from different species [191]. This is probably due to structural instability resulting from the mismatch between template and substrate. Although the mechanism by which distinct strains are generated remains unclear, distinct folds (templates) are formed from normal proteins, depending on genetic and/or environmental factors (Fig. 5a). Then, in the presence of a substrate matching the template (and cofactor), filaments composed of the same fold are formed and amplified (Fig. 5a). Disease-associated missense mutations directly affect the fibrous core structure and generate different strains. Structural analysis of mutant synthetic α-syn filaments associated with familial synucleinopathies has demonstrated that these mutations affect not only the core structure, but also the interface between the two protofilaments [36, 37, 122, 186, 279, 280, 323]. As environmental factors, the brain region and cell types (neurons, astrocytes and oligodendrocytes) in which aggregation initially occurs may contribute substantially to strain formation. Patient-derived pathogenic proteins have been shown experimentally to retain their properties even during amplification in different cellular environments [228]. The formation of 3R or 4R tau-specific strains in tauopathies may be caused by the heterogeneous expression of tau isoforms depending on the brain region and the cell type [54, 96, 195]. Oligodendrocyte-specific tubulin polymerization-promoting protein, p25α, is mislocalized to the cell body prior to α-syn accumulation in MSA cases, and it has been suggested that p25α may contribute to the formation of MSA-specific strains [96]. Differences in the aging process in each cell type may also be a factor. In addition, cell type-specific or non-specific cofactors could be directly responsible for the formation of disease-specific folds. Potential cofactors within the CTE and CBD folds include lipids and polyanions, which have been experimentally found to affect filament formation [86, 91, 97, 116, 322]. The involvement of PTMs in strain formation is supported by the combination of structural and mass analysis, which suggests that the ubiquitination at K340 that is characteristic of singlet-type CBD filaments prevents doublet formation [18]. As experimentally demonstrated, other proteins, lipids and nucleic acids interacting with prion-like proteins, salt concentration, pH and metals are also environment factors causing various forms of misfolding [12, 35, 57, 58, 117, 142, 150, 158, 270]. Once the filaments accumulate in the cell, the fragmented pathogenic seeds are released into the extracellular space and taken up by neighboring healthy cells. Although the mechanism of cell-to-cell transmission is not fully understood, the pathogenic seeds may be released through exocytosis or transport mediated by membrane vesicles, including synaptic vesicles and exosomes (Fig. 5b) [8, 60, 189, 217, 234, 254]. The leakage of pathogenic seeds from dead cells is also feasible. Extracellular pathogenic seeds may then be taken up into the cell directly, or by macropinocytosis, receptor-mediated endocytosis, or membrane fusion of exosomes (Fig. 5b) [31, 93, 100, 303]. Recently, it has also been demonstrated that pathogenic seeds can pass between neuronal cells via tunneling nanotubes (Fig. 5b) [2, 82, 285]. The continuous cell-to-cell transmission of these pathogenic proteins along the neuronal circuits leads to the intracerebral expansion of the pathology. A disease-specific fold is maintained during the amplification and spreading of pathogenic protein in the brain, resulting in the clinical and pathological diversity. Although the structures of α-syn filaments amplified by in vitro assays using brain and CSF samples derived from patients with synucleinopathies have been revealed by cryo-EM analysis, these structures are not identical to those of α-syn filaments extracted from patients’ brains [51, 190]. Therefore, the cellular environment and cofactors may play critical roles in strain formation.

## Directions for future research

The heterogeneity of abnormal proteins accumulated in patients’ brains is a key factor in the onset and progression of neurodegenerative diseases. Structural and biochemical analyses of post-mortem brains have revealed structural polymorphisms (strains) and prion-like properties of pathogenic proteins. Notably, recent cryo-EM studies of tau and α-syn filaments extracted from patients’ brains have provided direct evidence of strain formation in the brain (Table 1). Detailed structural information about these pathogenic proteins will contribute to the development of disease-modifying agents targeting protein aggregation and cell-to-cell transmission [261]. We believe there will be an increasing focus on the fibrous core structure in the design of aggregation inhibitors and antibodies for antibody therapy. The differences in the disease-specific fibrous core structures may also indicate that different approaches will be required for each disease. The same can be expected to apply to PET ligands used for early diagnosis, and this may lead to the development of PET ligands that can distinguish each disease within the same proteinopathy. Cryo-EM analysis has revealed the binding sites of EGCG, a natural compound that inhibits amyloid formation, and APN-1607, a PET ligand known as PM-PBB3, to tau filaments extracted from AD cases [260, 265]. Elucidation of the mechanisms underlying strain formation, as well as related factors, is crucial to reach a better understanding of the pathogenic protein strains in neurodegenerative diseases. The matured filaments that define each disease are supposed to pass through several different morphological forms during the maturation process. It will be important to clarify whether the disease-specific fold is established at the point of initial fibrillar species formation or at some other point during the maturation process, both in order to elucidate the mechanism of the strain formation and to characterize the pathological transition from pre-tangles/pre-inclusions to fibrillar inclusions. Recently, comprehensive proteomic analysis has identified a number of proteins that interact with tau, α-syn and TDP-43 [99, 123, 181, 292]. A similar approach would be useful to identify cell type-specific proteins that interact with pathogenic proteins and are involved in strain formation. In addition, the co-occurrence of multiple pathogenic proteins in the same patient is common in neurodegenerative diseases, but it is not fully understood how the interactions of these proteins contribute to the pathogenesis [146, 202]. Phosphorylated TDP-43 has been shown to co-localize in NFTs in AD and LBs in LBD, which may indicate a direct contribution of TDP-43 to AD and LBD pathogenesis [17, 138]. It has been also reported that distinct α-syn strains exhibit different abilities to induce tau aggregation and that the frequency of TDP-43 co-occurrence in tauopathies varies among diseases [124, 298]. The ways in which the interactions of multiple pathogenic proteins contribute to pathogenesis and the strain-specific characteristics of the interactions require further investigation. Structural analysis using cryo-EM will continue to be indispensable for the study of neurodegenerative diseases. It will be intriguing to see whether the abnormal proteins amplified in cellular and animal models inherit the structure of the patient-derived filaments used as the original seeds. In situ visualization of cultured cells and primary cultures with aggregates using cryo-EM tomography has also been reported, and analysis of brain samples from animals and patients using this approach may be fruitful [29, 126, 293]. Now that the importance of the cellular environment in strain formation has been suggested, understanding the changes in the cellular dynamics of cells with aggregates would be helpful to elucidate not only the mechanisms of aggregation, cell-to-cell transmission and neurodegeneration, but also the mechanism of strain formation.
