# Correction to: Ultrastructural and biochemical classification of pathogenic tau, α-synuclein and TDP-43

**DOI:** 10.1007/s00401-022-02439-y

**Published:** 2022-05-20

**Authors:** Airi Tarutani, Tadashi Adachi, Hiroyasu Akatsu, Yoshio Hashizume, Kazuko Hasegawa, Yuko Saito, Andrew C. Robinson, David M. A. Mann, Mari Yoshida, Shigeo Murayama, Masato Hasegawa

**Affiliations:** 1 Department of Brain and Neuroscience, Tokyo Metropolitan Institute of Medical Science, 2-1-6 Kamikitazawa, Setagaya-ku, Tokyo, 156-8506 Japan; 2 Division of Neuropathology, Department of Brain and Neurosciences, Faculty of Medicine, Tottori University, Tottori, 683-8503 Japan; 3 Department of Neuropathology, Choju Medical Institute, Fukushimura Hospital, Aichi, 441-8124 Japan; 4 Department of Community-Based Medical Education, Nagoya City University Graduate School of Medical Sciences, Aichi, 467-8601 Japan; 5 Division of Neurology, National Hospital Organization, Sagamihara National Hospital, Kanagawa, 252-0392 Japan; 6 Department of Neuropathology, Tokyo Metropolitan Institute of Gerontology, Tokyo, 173-0015 Japan; 7 Department of Pathology and Laboratory Medicine, National Center Hospital, National Center of Neurology and Psychiatry, Tokyo, 187-8551 Japan; 8 Faculty of Biology, Medicine and Health, School of Biological Sciences, Division of Neuroscience and Experimental Psychology, Salford Royal Hospital, The University of Manchester, Salford, M6 8HD UK; 9 Department of Neuropathology, Institute for Medical Science of Aging, Aichi Medical University, Aichi, 480-1195 Japan; 10 Brain Bank for Neurodevelopmental, Neurological and Psychiatric Disorders, United Graduate School of Child Development, Osaka University, Osaka, 565-0871 Japan

## Correction to: Acta Neuropathologica (2022) 143:613–640 10.1007/s00401-022-02426-3

In the sentence beginning “A C-shaped ….” in this article, the text “in 3R tau and G304-E380 in 4R tau” is mistakenly added. The correct sentence should read as “A C-shaped core structure consisting of V306-F378 was identified in the brain of one individual with AD.”

